# Assessment of cardiovascular regulation through irreversibility analysis of heart period variability: a 24 hours Holter study in healthy and chronic heart failure populations

**DOI:** 10.1098/rsta.2008.0265

**Published:** 2009-02-27

**Authors:** Alberto Porta, Gianni D'addio, Tito Bassani, Roberto Maestri, Gian Domenico Pinna

**Affiliations:** 1 Department of Technologies for Health, Galeazzi Orthopaedic Institute, University of Milan20161 Milan, Italy; 2 Fondazione S. Maugeri, IRCCS, Istituto di Riabilitazione di Telese82037 Telese, Italy; 3 Department of Bioengineering, Politecnico of Milan20133 Milan, Italy; 4 Fondazione S. Maugeri, IRCCS, Istituto di Riabilitazione di Montescano27040 Montescano, Italy

**Keywords:** heart rate variability, autonomic nervous system, time irreversibility, nonlinear dynamics, chronic heart failure, local prediction

## Abstract

We propose an approach based on time reversibility analysis to characterize the cardiovascular regulation and its nonlinearities as derived from 24 hours Holter recordings of heart period variability in a healthy population (*n*=12, age: median=43 years, range=34–55 years) and in a pathological group of age-matched chronic heart failure (CHF) patients (*n*=13, primarily in NYHA class II, age: median=37 years, range=33–56 years, ejection fraction: median=25%, range=13–30%). Two indices capable of detecting nonlinear irreversible dynamics according to different strategies of phase-space reconstruction (i.e. a fixed two-dimensional phase-space reconstruction and an optimal selection of the embedding dimension, respectively) are tested and compared with a more traditional nonlinear index based on local nonlinear prediction. Results showed that nonlinear dynamics owing to time irreversibility at short time scales are significantly present during daytime in healthy subjects, more frequently present in the CHF population and less frequently during night-time in both groups, thus suggesting their link with a dominant sympathetic regulation and/or with a vagal withdrawal. On the contrary, nonlinear dynamics owing to time irreversibility at longer, dominant time scales were insignificantly present in both groups. During daytime in the healthy population, irreversibility was mostly due to the presence of asymmetric patterns characterized by bradycardic runs shorter than tachycardic ones. Nonlinear dynamics produced by mechanisms different from those inducing temporal irreversibility were significantly detectable in both groups and more frequently during night-time. The present study proposes a method to distinguish different types of nonlinearities and assess their contribution over different temporal scales. Results confirm the usefulness of this method even when applied in uncontrolled experimental conditions such as those during 24 hours Holter recordings.

## 1. Introduction

Short beat-to-beat cardiovascular series (approx. 300 beats or 5 min) are routinely processed to derive indirect indices assessing short-term cardiovascular regulation carried out through neural and non-neural mechanisms (Cohen & Taylor 2002). Short series are mostly assessed by methods hypothesizing linear dynamics such as spectral analysis (Akselrod *et al*. 1981; Malliani *et al*. 1991) or multivariate parametric modelling (Xiao *et al*. 2005; Porta *et al*. 2006), thus preventing to account for the possible presence of nonlinear dynamics. In addition, when nonlinear methods capable of dealing with the short cardiovascular series (Pincus 1995; Voss *et al*. 1996, 2009; Wessel *et al*. 2000; Porta *et al*. 2000, 2009) were applied, these techniques were able to detect nonlinear dynamics, but failed to provide more specific information about the type of nonlinear mechanism capable of producing them. Time irreversibility analysis can detect a more specific type of nonlinear dynamics (Weiss 1975) capable of producing temporal asymmetries resulting in statistical properties that are different when the series are observed after time reversal.

The time course of heart period commonly exhibits short-term time irreversibility (Braun *et al*. 1998; Costa *et al*. 2005; Guzik *et al*. 2006; Porta *et al*. 2008), and irreversibility analysis provides important information about cardiovascular control (Porta *et al*. 2008). The importance of time irreversibility in heart period series was also suggested by studies that did not explicitly interpret their findings in terms of time irreversibility (see Ohashi *et al*. 2003). However, it is unclear whether nonlinear mechanisms producing time irreversibility are the exclusive nonlinear mechanisms producing the nonlinear dynamics present in heart period series.

Functionals traditionally used to detect time irreversibility have an important drawback. They are devised for signals generated by low-dimensional systems. Indeed, they are typically based on two-dimensional phase-space reconstruction of the dynamics (Elhers *et al*. 1998; Costa *et al*. 2005; Guzik *et al*. 2006; Porta *et al*. 2008), thus failing in the presence of high-dimensional dynamics that are roughly unfolded in a plane. Moreover, when functionals are not restricted to work in a two-dimensional phase space (Diks *et al*. 1995), no strategy has been provided for fixing the embedding dimension, thus leaving the arbitrary selection of the phase-space dimension as the sole viable possibility (Stone *et al*. 1996; van der Heyden *et al*. 1996; Hoekstra *et al*. 1997).

The aim of this study is threefold. First is to assess the information provided by time irreversibility analysis of short beat-to-beat heart period sequences derived from 24 hours Holter recordings in healthy and pathological populations. To address this aim, we contrast a group of heart failure patients with a healthy group. Second is to assess the information that can be derived from the exploration of time irreversibility in higher dimensional phase spaces. To deal with this aim, we propose a new index, i.e. the normalized difference between backward and forward unpredictability indices (FBUPI), based on an optimal selection of the embedding dimension and capable of detecting irreversible dynamics that cannot be revealed using simpler indices based on two-dimensional phase-space reconstruction. Third is to compare the percentage of nonlinear dynamics, as detected by means of a test based on a local nonlinear prediction approach, with that detected by time irreversibility analysis. Since irreversible dynamics are a subset of the entire collection of nonlinear dynamics, this comparison may allow understanding of the importance of nonlinear mechanisms producing time irreversibility with respect to those generating more generic nonlinear dynamics.

## 2. Detecting nonlinear dynamics

We considered two indices capable of detecting time irreversibility and one index capable of identifying more generic nonlinear behaviours. The two time irreversibility indices are the fraction of negative variations (appendix A*b*) and FBUPI (see appendix A*c*). The forward unpredictability index (i.e. FUPI, see appendix A*c*) was used to detect more generic nonlinear dynamics.

We used a surrogate data approach to detect nonlinearity and irreversibility. We set as a null hypothesis that the series was a linear Gaussian process possibly distorted via a nonlinear static invertible transformation. This null hypothesis is helpful for detecting basic dynamical nonlinearities, when FUPI is used, and irreversibility, when N% or FBUPI are exploited. Accordingly, we built surrogate series with preserved power spectrum and the same distribution as the original ones. Iteratively refined amplitude-adjusted Fourier transform (IAAFT) surrogates were constructed according to Schreiber & Schmitz (1996). The maximum number of iterations was set to 100. We constructed a set of 250 IAAFT surrogates for each original sequence. The parameters N%, FUPI and FBUPI were calculated over the surrogate series (N%_s_, FUPI_s_ and FBUPI_s_) and over the original series (N%_o_, FUPI_o_ and FBUPI_o_). We implemented the following two-sided non-parametric tests over N%, FUPI and FBUPI.

If N%_o_ (or FBUPI_o_) was smaller than the 2.5th percentile of the N%_s_ (or FBUPI_s_) distribution or larger than the 97.5th percentile, the null hypothesis of reversibility was rejected and the original series was said to be irreversible. If N%_o_ was larger than the 97.5th percentile of the N%_s_ distribution, the number of negative variations was significantly larger than that of positive variations. In the case of FBUPI, FBUPI_o_ larger than the 97.5th percentile of the FBUPI_s_ distribution indicated that the forward prediction was significantly better than the backward one. N%_o_ (or FBUPI_o_) smaller than the 2.5th percentile of the N%_s_ (or FBUPI_s_) distribution indicated the complementary type of irreversible dynamics.

If FUPI_o_ was smaller than the 2.5th percentile of the FUPI_s_ distribution, the null hypothesis of linearity was rejected (i.e. the original series was predicted forward better than the linear surrogates) and the original series was said to be nonlinear.

## 3. Simulations

### (a) Linear processes

Autoregressive (AR) processes driven by white Gaussian noise were used to simulate linear, reversible, partially predictable processes, thus allowing the assessment of the percentage of the erroneous rejections of the null hypothesis of linear, reversible dynamics. AR processes were shaped to exhibit oscillations at the same frequency of those usually found in short-term heart period variability series (i.e. rhythms in the low-frequency (LF) band at approx. 0.1 cycles per beat and in the high-frequency (HF) band at approx. 0.25 cycles per beat). Therefore, two types of AR processes were considered: (i) a second-order AR process (AR(2)) with two complex and conjugated poles with phases *φ*=±0.1 cycles per beat termed as AR(2)_LF_, and (ii) an AR(2) process with two complex and conjugated poles with phases *φ*=±0.25 cycles per beat termed as AR(2)_HF_. In both AR(2)_LF_ and AR(2)_HF_ processes, the pole modulus, *ρ*, was varied from 0.77 to 0.98 according to steps of 0.03, thus increasing the sharpness of the spectral peak and the regularity of the process, thus allowing the assessment of the effects of changes in the process regularity on the percentage of the erroneous rejections of the null hypothesis of linear, reversible dynamics. Assigning *φ* and *ρ*, we generated 20 different AR(2) realizations. Series were normalized to have zero mean and unit variance. In the case of AR(2)_LF_, these realizations will be labelled as L77, …, L98, while in the case of AR(2)_HF_ as H77, …, H98.

### (b) Nonlinear processes

The delayed tent map (DT) described by *x*(*i*+1)=2*kx*(*i*−*σ*) for 0<*x*(*i*−*σ*)<0.5 and *x*(*i*+1)=2*k*(1−*x*(*i*−*σ*)) for 0.5<*x*(*i*−*σ*)<1 was used to generate nonlinear deterministic processes bounded between 0 and 1, where *σ* represents the delay of the tent map, thus allowing the assessment of the percentage of the correct rejections of the null hypothesis of linear, reversible dynamics. The value of *k* was set to produce chaotic dynamics (*k*=0.9) with *σ*=0. Two types of simulations were considered: (i) DT with *σ*=0 termed as DT0 and (ii) DT with *σ*=1 termed as DT1. Both DT0 and DT1 generate irreversible dynamics, but, while in the case of DT0 irreversibility can be detected using a two-dimensional phase-space reconstruction of the dynamics, in the case of DT1 a higher dimensional phase-space reconstruction is needed to detect irreversibility (Casali *et al*. 2008). DT realizations were rescaled to have zero mean and unit variance. In order to blur nonlinear dynamics, we summed a zero mean Gaussian white noise with assigned variance (it was 0.05, 0.5, 1.0 and 1.5) to the DT realizations. In the case of DT0, these realizations will be labelled as DT0_05, DT0_50, DT0_100 and DT0_150, while in the case of DT1 as DT1_05, DT1_50, DT1_100 and DT1_150, thus allowing the assessment of the effects of noise on the percentage of the correct rejections of the null hypothesis of linear, reversible dynamics.

## 4. Experimental protocol and data analysis

### (a) Experimental protocol

We studied 12 healthy normal (NO) subjects (age: median=43 years, range=34–55 years) and 13 age-matched chronic heart failure (CHF) patients (two were in NYHA class I, two in NYHA class III and the remaining in class II, age: median=37 years, range=33–56 years, ejection fraction: median=25%, range=13–30%). They underwent a 24 hours Holter recording (Oxford Medilog System) during usual everyday activities (Maestri *et al*. 2007). Sampling frequency was 250 Hz. Each beat was automatically labelled as normal or aberrant by the Holter analysis software and, then, carefully edited by an expert analyst. Heart period was approximated as the temporal distance between two consecutive R peaks. Twenty-four hour beat-to-beat RR series were pre-processed according to the criteria reported in Maestri *et al*. (2007).

### (b) Data analysis of 24 hours Holter heart period recordings

The 24 hours heart period variability series were analysed during daytime (from 09.00 to 19.00 hours) and night-time (from 00.00 to 05.00 hours). Irreversibility analysis was applied to sequences of 256 cardiac beats with a 40 per cent overlap. Only recordings with at least 50 per cent of the analysed periods in sinus rhythm during both night- and daytime were considered. The time series were linearly detrended. During both day- and night-time, we constructed the distribution of N% and FBUPI. The median of the distribution was extracted for successive statistical analyses. For each 24 hours Holter recording, we calculated the percentage of nonlinear dynamics, NL_FUPI_, and the percentage of irreversible series, I_N%_ and I_FBUPI_. Moreover, we evaluated the percentage of irreversible dynamics in which the number of negative variations was significantly larger than that of positive variations indicated by ${\text{I}}_{\text{N}\%}^{+}$, and that with the forward prediction better than the backward one, indicated by ${\text{I}}_{\text{FBUPI}}^{+}$. Results are reported as mean±s.d. of all the subjects.

### (c) Statistical analysis

Paired *t*-test (or Wilcoxon signed-rank test when appropriate) was used to test the difference between parameters derived during day- and night-time inside the same population (NO or CHF). Unpaired *t*-test (or Mann–Whitney rank-sum test when appropriate) was carried out to check the difference between parameters derived from NO and CHF populations inside the same period of analysis (day- or night-time). *p*<0.05 was considered statistically significant.

## 5. Results

### (a) Simulation results

**Figure 1:**
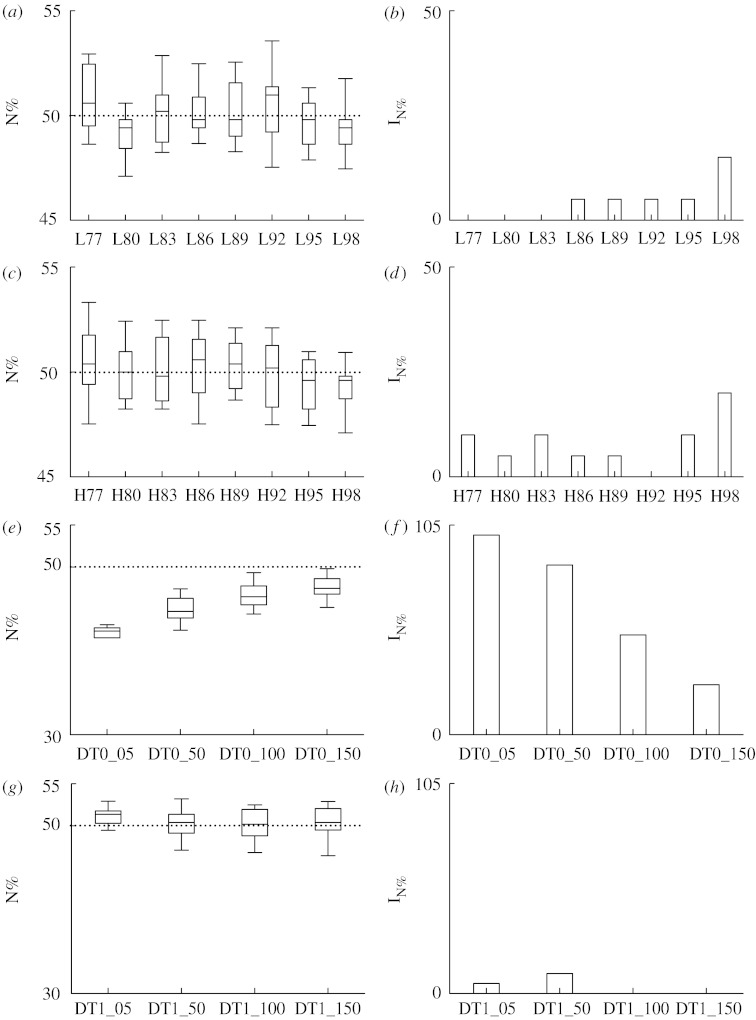
(*a*,*c*,*e*,*g*) Box-and-whisker plots relevant to N% and (*b*,*d*,*f*,*h*) bar graphs relevant to the percentage of irreversible dynamics, I_N%_, as derived from 20 realizations of (*a*,*b*) AR(2)_LF_, (*c*,*d*) AR(2)_HF_, (*e*,*f*) DT0 and (*g*,*h*) DT1 processes. Indices N% and I_N%_ are assessed as a function of the pole modulus in the case of AR(2)_LF_ and AR(2)_HF_ and as a function of the amplitude of the additive reversible noise in the case of DT0 and DT1.

Figure 1 reports the results relevant to N% as derived from simulated signals. Box-and-whisker plots (figure 1*a*,*c*,*e*,*g*) report the 10th, 25th, 50th, 75th and 90th percentiles of N% as calculated over 20 realizations of the simulated series (i.e. AR(2)_LF_, AR(2)_HF_, DT0 and DT1), while the bar graphs (figure 1*b*,*d*,*f*,*h*) show the percentage of irreversible dynamics I_N%_. The index N% tended to remain at approximately 50 in AR(2)_LF_ and AR(2)_HF_ realizations (figure 1*a*,*c*, respectively), thus suggesting that, as theoretically expected, the null hypothesis of reversibility was unlikely to be rejected. This feeling was confirmed by the surrogate approach: indeed, I_N%_ detected over 20 realizations of AR(2)_LF_ and AR(2)_HF_ (figure 1*b*,*d*, respectively) was close to 0 and reached 15 and 20 only in the case of AR(2)_LF_ and AR(2)_HF_ series with *ρ*=0.98 (i.e. L98 and H98). The index N% was significantly smaller than 50 when assessed over DT0 blurred by low levels of noise (i.e. DT0_05) and tended to increase towards 50 as a function of the relevance of the additive noise (figure 1*e*). Surrogate data analysis proved that, when the amplitude of the reversible noise obscuring irreversible tent map dynamics was low, I_N%_ was 100 and it progressively decreased as a function of the amplitude of noise (figure 1*f*). On the contrary, when DT1 realizations were considered, N% was approximately 50 independently of the amplitude of the noise blurring tent map dynamics (figure 1*g*), suggesting the inability of N% in detecting the displacement of irreversibility towards higher dimensions produced by a delay different from 0. Accordingly, I_N%_ was close to 0 even when the amount of reversible noise blurring the tent map dynamics was small (figure 1*h*).

**Figure 2:**
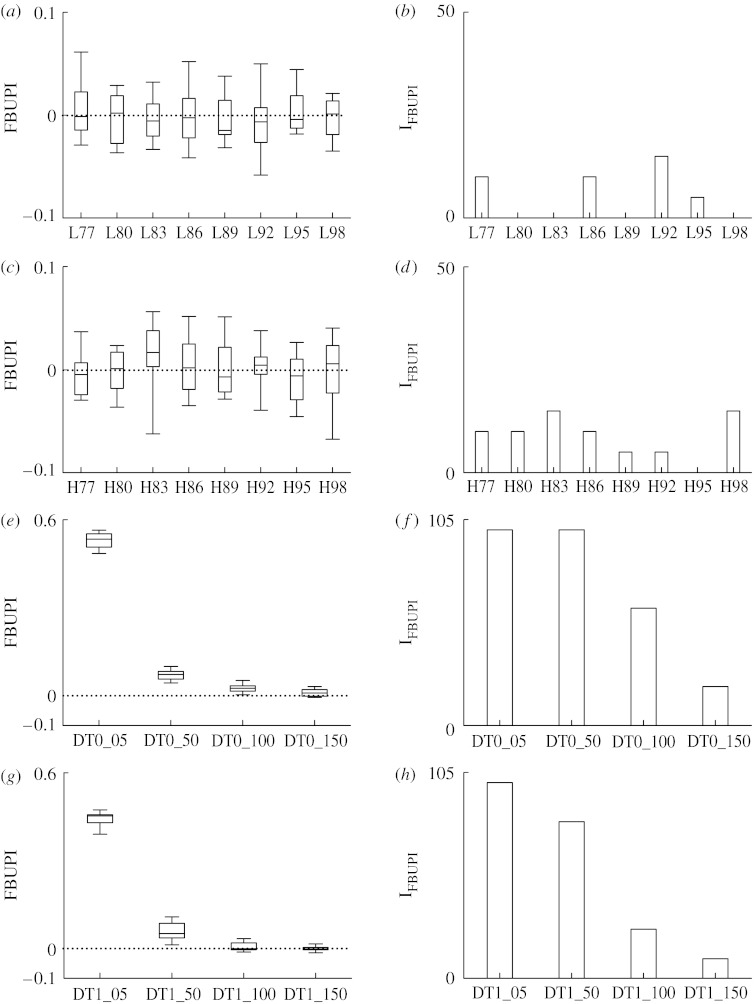
(*a*,*c*,*e*,*g*) Box-and-whisker plots relevant to FBUPI and (*b*,*d*,*f*,*h*) bar graphs relevant to the percentage of irreversible dynamics, I_FBUPI_, as derived from 20 realizations of (*a*,*b*) AR(2)_LF_, (*c*,*d*) AR(2)_HF_, (*e*,*f*) DT0 and (*g*,*h*) DT1 processes. Indices FBUPI and I_FBUPI_ are assessed as a function of the pole modulus in the case of AR(2)_LF_ and AR(2)_HF_ and as a function of the amplitude of the additive reversible noise in the case of DT0 and DT1.

Results relevant to the application of FBUPI to simulated signals are shown in figure 2, which has the same structure as figure 1. The index FBUPI remained at approximately 0 in AR(2)_LF_ and AR(2)_HF_ simulations (figure 2*a*,*c*, respectively), thus suggesting that, as theoretically expected in reversible signals, the forward prediction was not better than the backward one and vice versa. The surrogate approach corroborated this impression: indeed, the percentage of irreversible dynamics, I_FBUPI_, derived from 20 realizations of AR(2)_LF_ and AR(2)_HF_ processes (figure 2*b*,*d*, respectively) was close to 0 and reached 15 only in the case of AR(2)_LF_ series with *ρ*=0.92 (i.e. L92) and in the case of AR(2)_HF_ ones with *ρ*=0.83 and 0.98 (i.e. H83 and H98). The index FBUPI was significantly larger than 0 when assessed over DT0 blurred by low levels of noise (i.e. DT0_05) and tended to decrease towards 0 as a function of the amplitude of the additive noise (figure 2*e*). Surrogate data analysis proved that I_FBUPI_ was 100 even when the amplitude of the additive reversible noise was remarkable as in DT0_50 series and decreased in the presence of higher levels of noise (figure 2*f*). It is noteworthy that similar trends of FBUPI and I_FBUPI_ were observed in the case of DT1 realizations (figure 2*g*,*h*), thus indicating that FBUPI was capable of coping with the displacement of irreversibility towards higher dimensions produced by a delay equal to 1.

**Figure 3:**
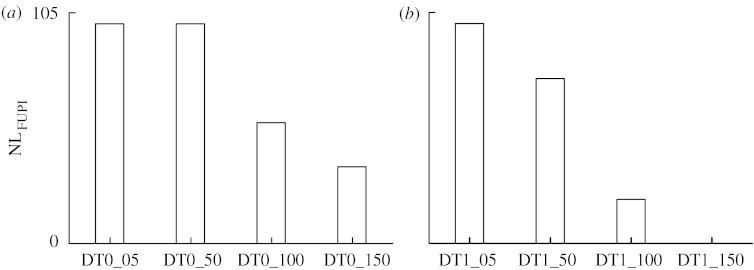
The percentage of nonlinear dynamics, NL_FUPI_, as derived from 20 realizations of (*a*) DT0 and (*b*) DT1 processes as a function of the amplitude of the additive reversible noise.

The percentage of nonlinear dynamics as detected by FUPI, NL_FUPI_, derived from 20 realizations of DT0 and DT1 series exhibited trends (figure 3*a*,*b*) similar to those reported by figure 2*f*,*h*, thus indicating that nonlinearities different from those resulting from temporal irreversibility were not present in DT0 and DT1 dynamics.

### (b) Experimental results

**Figure 4:**
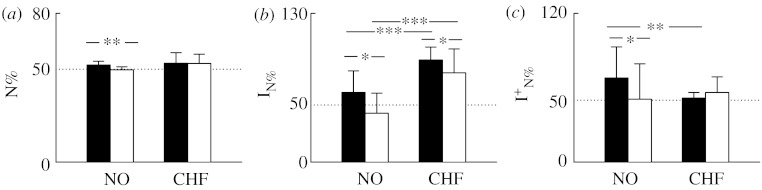
Bar graphs show (*a*) N%, (*b*) the percentage of irreversible dynamics, I_N%_, and (*c*) the percentage of irreversible dynamics with the number of negative variations significantly higher than that of positive variations, ${\text{I}}_{\text{N}\%}^{+}$, derived from NO subjects and CHF patients during daytime (filled bars) and night-time (open bars). ^***^p<0.001, ^**^p<0.01 and ^*^p<0.05.

**Figure 5:**
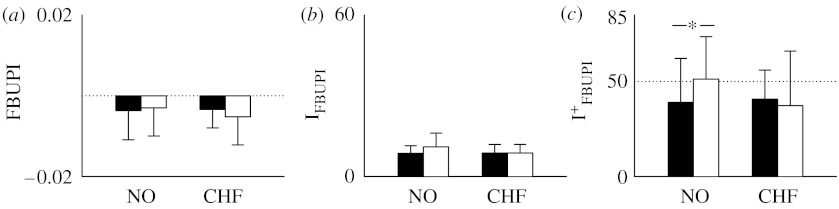
Bar graphs show (*a*) FBUPI, (*b*) the percentage of irreversible dynamics, I_FBUPI_, and (*c*) the percentage of irreversible dynamics that can be predicted forwards better than backwards, and ${\text{I}}_{\text{FBUPI}}^{+}$, derived from NO subjects and CHF patients during daytime (filled bars) and night-time (open bars). ^*^p<0.05.

**Figure 6:**
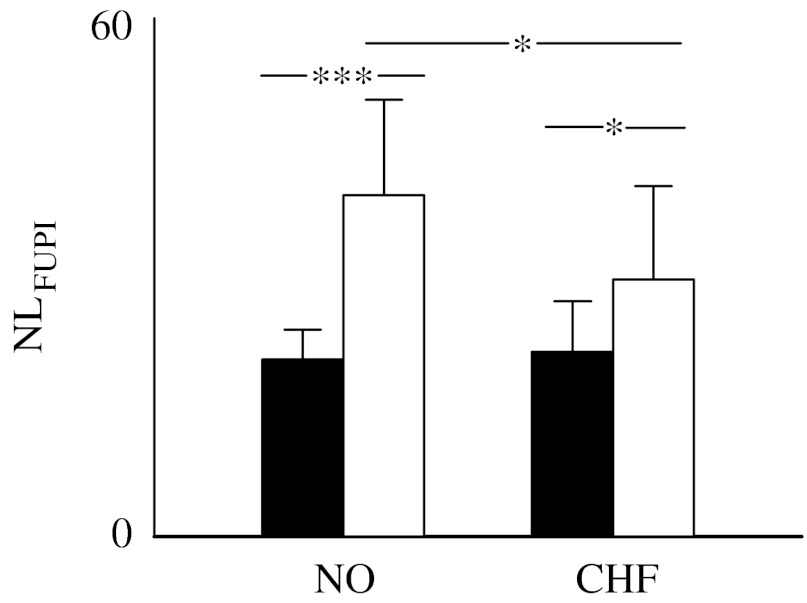
The bar graph shows the percentage of nonlinear dynamics, NL_FUPI_, detected by FUPI and derived from NO subjects and CHF patients during daytime (filled bars) and night-time (open bars). ^***^p<0.001 and ^*^p<0.05.

Results of irreversibility analysis as derived during day- and night-time in NO subjects and CHF patients are shown in figures 4–6.

Results relevant to N% are depicted in figure 4. Bar graphs show the mean (+s.d.) of N% (figure 4*a*), I_N%_ (figure 4*b*) and ${\text{I}}_{\text{N}\%}^{+}$ (figure 4*c*) during daytime (filled bars) and night-time (open bars) in NO subjects and CHF patients. In NO subjects during daytime, N% (figure 4*a*) was slightly larger than 50 (i.e. 52) and significantly decreased during night-time. In CHF patients, N% (figure 4*a*) tended to be larger during daytime (i.e. 53) and remained unchanged during night-time. The index I_N%_ (figure 4*b*) was high in NO subjects during daytime (i.e. 61) and decreased significantly during night-time (i.e. 43). In CHF patients, I_N%_ was significantly higher than that in NO subjects during both day- and night-time (figure 4*b*). In addition, in CHF patients, the day–night variation of I_N%_ was preserved (figure 4*b*). Irreversible dynamics detected during daytime in NO subjects were mostly due to bradycardic runs shorter than tachycardic ones (i.e. ${\text{I}}_{\text{N}\%}^{+}=68$; figure 4*c*). Their presence in NO subjects was significantly reduced during night-time (${\text{I}}_{\text{N}\%}^{+}=51$; figure 4*c*). In the CHF population during daytime, ${\text{I}}_{\text{N}\%}^{+}$ was significantly less frequent than that in NO subjects and the circadian variation disappeared (figure 4*c*).

Results derived from FBUPI are depicted in figure 5. Bar graphs show the mean of FBUPI (figure 5*a*), I_FBUPI_ (figure 5*b*) and ${\text{I}}_{\text{FBUPI}}^{+}$ (figure 5*c*) derived during daytime (filled bars) and night-time (open bars) in NO subjects and CHF patients. Both in NO subjects and CHF patients, the optimal embedding dimension was more likely detected at 3 and 4 during day- and night-time, respectively, independently of the prediction method (i.e. forward or backward). FBUPI was slightly negative indicating that the backward prediction was slightly better than the forward one independently of the considered population (figure 5*a*). In addition, no circadian variation was observable (figure 5*a*). The small difference between forward and backward prediction was not sufficient to detect a significant amount of irreversible series (figure 5*b*): indeed, I_FBUPI_ was approximately 10 independently of population and period of analysis. Among the small amount of irreversible dynamics, those with the forward prediction better than the backward one (figure 5*c*) were less frequent than the complementary ones (i.e. ${\text{I}}_{\text{FBUPI}}^{+}$ is below 50) in the NO population during daytime and the situation was more balanced during night-time. In CHF patients, ${\text{I}}_{\text{FBUPI}}^{+}$ was similar to that in NO subjects during daytime.

Figure 6 shows the percentage of nonlinear dynamics as detected by FUPI (i.e. NL_FUPI_). NL_FUPI_ was small in NO subjects during daytime and increased significantly during night-time (i.e. 20 versus 40). The same significant trend was observed in CHF patients, even though the increase during night-time was smaller (i.e. 21 versus 30). It is noteworthy that NL_FUPI_ was significantly larger than I_FBUPI_ (figure 5*b*), but smaller than I_N%_ (figure 4*b*) independently of the population and period of analysis.

## 6. Discussion

### (a) Irreversibility indices and embedding dimension

The major methodological findings of this study are as follows: (i) both indices N% and FBUPI are characterized by a low rate of erroneous rejections of the null hypothesis of reversibility, almost independent of the regularity of the process, and by a high rate of correct rejections of the null hypothesis of reversibility even in the presence of a large amount of additive reversible noise corrupting irreversible dynamics, (ii) the index based on a two-dimensional phase-space reconstruction of the dynamics (i.e. N%) cannot detect the displacement of irreversibility towards higher dimensions, and (iii) the index that does not fix *a priori* the embedding dimension (i.e. FBUPI) is more suitable to deal with the issue of the displacement of time irreversibility towards higher dimensions, while keeping low the rate of erroneous rejections of the null hypothesis of reversibility.

This study has assessed the performance of two indices capable of detecting time irreversibility: the percentage of negative variations (i.e. N%) and the normalized difference between forward and backward unpredictability indices (i.e. FBUPI) derived from a local nonlinear prediction approach. These indices differ in the strategy of the phase-space reconstruction. Indeed, while N% is devised according to a two-dimensional phase-space reconstruction, FBUPI is obtained without fixing *a priori* the embedding dimension but, conversely, by exploiting the embedding dimension most helpful to predict the series in the forward and backward temporal directions. In agreement with these differences, N% and FBUPI exhibit different capabilities in detecting irreversible dynamics.

The tent map in the chaotic regime produces irreversible dynamics that are characterized by an asymmetry of the dominant pattern (i.e. the downward side of the pattern is steeper than the upward one) and are largely predictable, although incompletely, from past values but completely unpredictable using future samples. In agreement with these observations, we found that N% was significantly smaller than 50 (figure 1*e*) and FBUPI significantly larger than 0 (figure 2*e*). It is noteworthy that both N% and FBUPI could detect irreversible dynamics produced by the tent map in the chaotic regime even in the presence of a significant amount of noise (figures 1*f* and 2*f*). These performances are outstanding when taking into account that the rate of erroneous rejections of the null hypothesis of reversibility, as measured by considering AR realizations, remains quite limited and almost independent of the dominant oscillation frequency and spectral peak sharpness. Indeed, we observed a slight increase in erroneous rejections only in the case of faster oscillations and/or in the case of very sharp peak produced by poles very close to the instability region, i.e. the unit circle (figures 1*b*,*d* and 2*d*).

The DT produces a displacement of temporal irreversibility towards higher dimensions (Casali *et al*. 2008). Therefore, too simple indices based on two-dimensional phase-space reconstruction, such as N%, cannot detect it (figure 1*g*,*h*) and more complex indices, such as FBUPI, based on higher dimensional phase-space reconstruction are necessary (figure 2*g*,*h*). It is noteworthy that FBUPI has the advantage of detecting higher dimensional irreversible dynamics produced by the DT without increasing the fraction of false positives as happens when using the multiple test strategy proposed in Casali *et al*. (2008).

Given the results of simulations, we can conclude that FBUPI is better than N% since it exhibits good performances when applied to both tent and DT maps; it is reliable in the presence of additive reversible noise and keeps the rate of erroneous rejections of the null hypothesis of reversibility at an acceptable level. It is also important to stress that the comparison of the percentage of nonlinear dynamics, as detected by the forward local nonlinear prediction approach (i.e. by FUPI), and the percentage of nonlinear dynamics owing to time irreversibility, as detected by FBUPI, are similar (figure 2*f*,*h* versus figure 3*a*,*b*), thus indicating that the nonlinear mechanisms producing irreversible dynamics are responsible for the full amount of nonlinear dynamics detectable in the series.

### (b) Irreversibility indices, nonlinear dynamics and cardiovascular control

As to the application to cardiovascular control in healthy and pathological groups, the major findings are as follows: (i) the percentage of irreversible dynamics as detected by a simple index based on two-dimensional phase-space reconstruction (i.e. N%) is significant in NO subjects during daytime and decreases during night-time, (ii) irreversible sequences as detected by N% are more likely in CHF patients and circadian variation is preserved, (iii) in NO subjects during daytime irreversibility is mostly due to the presence of bradycardic runs shorter than tachycardic ones (i.e. the heart decelerates more quickly than it accelerates) and this pattern is less frequent during night-time and in the CHF population, (iv) the percentage of irreversible dynamics as detected by an index based on the optimization of the dimension of the phase-space reconstruction (i.e. FBUPI) is marginal both in NO and CHF subjects and does not exhibit any circadian variation, (v) the percentage of nonlinear dynamics as detected by an index based on the optimization of the embedding dimension (i.e. FUPI) is smaller than the percentage of irreversible dynamics as detected by N% and higher than that as detected by FBUPI, and (vi) the percentage of nonlinear dynamics detected by FUPI increases during night-time and the increase is less remarkable in the CHF population.

We confirm that, when assessed using a simple index based on two-dimensional phase-space reconstruction (i.e. N%) such as those used in previous work (Braun *et al*. 1998; Costa *et al*. 2005; Guzik *et al*. 2006; Porta *et al*. 2008), RR variability is irreversible in NO humans even during uncontrolled experimental conditions such as those during 24 hours Holter recordings (figure 4*b*). Previous studies indicated that, under controlled experimental conditions in NO subjects, temporal asymmetries responsible for the irreversibility of short-term RR variability are present at rest in the supine position and are more frequent during sympathetic activation induced by head-up tilt test especially at the highest degrees of tilt table inclination (Porta *et al*. 2008). The present study confirms the link between the autonomic nervous system and irreversibility of RR series: indeed, irreversible RR sequences are more likely during daytime when sympathetic activity is higher. Also, the reduced presence of irreversible dynamics during night-time (figure 4*b*) can be ascribed to the sympathetic withdrawal and/or the vagal enhancement occurring during night-time, thus again suggesting the key role of the autonomic nervous system in causing temporal asymmetries responsible for irreversibility.

We confirm the link between irreversible dynamics as detected by a simple index based on two-dimensional phase-space reconstruction and pathology already observed in biomedical fields different from the cardiovascular one (Timmer *et al*. 1993; van der Heyden *et al*. 1996). Indeed, we observed a higher percentage of irreversible RR dynamics in CHF patients during both day- and night-time (figure 4*b*), thus again suggesting the link between irreversibility and sympathetic regulation that is well known to be overexpressed in CHF patients. However, pathology did not disrupt the circadian pattern: indeed, in CHF patients the day–night variation was still preserved (figure 4*b*). These results are in disagreement with those reported by Costa *et al*. (2005). Further studies are needed to understand whether this disagreement is the result of the method proposed by Costa *et al*. (2005) merging together multiple temporal scales or of the lack of a rigorous case-by-case comparison with surrogate data.

We found that time irreversibility of heart period variability in NO subjects during daytime is mostly due to the appearance of bradycardic runs shorter than tachycardic ones (i.e. the heart decelerates more rapidly than it accelerates), while the reverse pattern (tachycardic runs shorter than bradycardic ones) is less present (figure 4*c*). The same conclusion was drawn when assessing short-term heart period variability in well-controlled experimental conditions (Porta *et al*. 2008). It is noteworthy that a more balanced situation was found in NO subjects during night-time, thus suggesting that the autonomic nervous system might modulate the proportions of these two complementary forms of temporal asymmetries. A balanced presence between patterns with bradycardic runs shorter than tachycardic ones and reversed features was observed in CHF patients independently of the period of the analysis (figure 4*c*). Therefore, in CHF patients, the increased presence of irreversible dynamics was accompanied by an important change in the relative proportion of the two different patterns responsible for this peculiar type of nonlinear dynamics.

When irreversibility was detected with an index based on the optimization of the phase-space embedding dimension (i.e. FBUPI), the percentage of irreversible dynamics was negligible with respect to that detected using an index based on two-dimensional phase-space reconstruction (i.e. N%; figure 5*b* versus figure 4*b*). This result can be explained by considering that FBUPI tends to focus on dominant, longer time scales, while N% tends to focus on shorter ones, thus suggesting the presence of different degrees of irreversibility as a function of the time scales.

Since the detection of time irreversibility implies the presence of nonlinear dynamics (Weiss 1975), results derived from N% suggest that nonlinearities are important in NO subjects during daytime, are more relevant in the CHF population and their importance decreases in both populations during night-time. Since N% is more sensible to short temporal scales, we can conclude that heart period variability is significantly nonlinear over short temporal scales both in NO and CHF populations and the importance of these nonlinearities decreases during night-time. It is noteworthy that the percentage of nonlinear dynamics as derived from an index based on local forward nonlinear prediction (i.e. FUPI) provides different results (figure 6). Indeed, the percentage of nonlinear dynamics is significantly smaller than the percentage of irreversible dynamics derived by N%. In addition, the percentage of nonlinear dynamics detected by FUPI increases during night-time, while the percentage of irreversible dynamics as detected by N% decreases. Results derived from FUPI are in line with previous studies that found a higher percentage of nonlinear dynamics under experimental conditions imposing regular breathing patterns and a smaller percentage of nonlinear dynamics under experimental conditions characterized by an important sympathetic modulation (Porta *et al*. 2000, 2007). This seeming contradiction can be solved by considering that FUPI is derived after the optimization of the embedding dimension, thus focusing on dominant, longer time scales, while N% focuses on fast time scales. Therefore, we suggest that dominant, longer temporal scales are less characterized by nonlinear dynamics than shorter ones and become nonlinear when respiratory sinus arrhythmia is important and respiration is more regular (Porta *et al*. 2000, 2007). This finding stresses the importance of accounting for the time-scale focalization of indices used to detect nonlinear dynamics when interpreting the final results. In addition, when two indices focusing on longer, dominant time scales were compared (i.e. FBUPI and FUPI), it was found that the percentage of nonlinear dynamics owing to temporal irreversibility as detected by FBUPI is smaller than that of more generic nonlinear dynamics as detected by FUPI (figure 5*b* versus figure 6), thus stressing the importance of the proposed approach to differentiate different types of nonlinear dynamics.

## 7. Conclusions

Time irreversibility parameters are useful to characterize the physiology of cardiovascular control during daily activities both in healthy and pathological populations. These parameters may stimulate a more insightful interpretation of the cardiovascular regulation by suggesting the temporal correlates for the nonlinear behaviour, the development of more precise models of short-term cardiac control including specific nonlinearities at their corresponding time scales, the generation of more faithful synthetic heart period series and the design of clinical and pharmacological studies to clarify the mechanisms underlying the observed behaviour. In addition, the proposed approach provides a tool to differentiate nonlinear dynamics owing to time irreversibility from less specific nonlinear dynamics and to derive information associated with temporal scales.

## Appendix A

### (a) General definitions

Given the stationary series *x*={*x*(*k*), *k*=1, …, *N*}, let us define $x_{L}^{\text{past}}(i)=(x(i),x(i-1),\ldots ,x(i-L+1))$ the pattern constituted by the current sample *x*(*i*) and by the pattern $x_{L-1}^{\text{past}}(i-1)$ containing *L*−1 past samples of *x*(*i*) $\left(\text{i.e.}\;x_{L-1}^{\text{past}}(i-1)=(x(i-1),\ldots ,x(i-L+1))\right)$. Let us now consider the pattern $x_{L}^{\text{future}}(i)=(x(i),x(i+1),\ldots ,x(i+L-1))$ constituted by the current sample *x*(*i*) and by the pattern $x_{L-1}^{\text{future}}(i+1)$ containing *L*−1 future samples of *x*(*i*) $\left(\text{i.e.}\;x_{L-1}^{\text{future}}(i+1)=(x(i+1),\ldots ,x(i+L-1))\right)$. When helpful in the following, the patterns $x_{L}^{\text{past}}(i)$ and $x_{L}^{\text{future}}(i)$ will be indicated as $x_{L}^{\text{past}}(i)=(x(i),x_{L-1}^{\text{past}}(i-1))$ and $x_{L}^{\text{future}}(i)=(x(i),x_{L-1}^{\text{future}}(i+1))$, respectively, thus stressing that *x*(*i*) may be predicted forwards using the past values, $x_{L-1}^{\text{past}}(i-1)$, or backwards using the future values, $x_{L-1}^{\text{future}}(i+1)$.

Let us define as *x*^*r*^={*x*^*r*^(*k*)=*x*(*N*−*k*+1), *k*=1, …, *N*} the series obtained from *x* after time reversal. It might be helpful in the following to keep in mind the following result that sets the equivalence between backward prediction of *x* and forward prediction on *x*^*r*^. Indeed, owing to the relationship between *x* and *x*^*r*^, the pattern $x_{L}^{r,\text{past}}(i)=(x^{r}(i),x_{L-1}^{r,\text{past}}(i-1))$ is formed by the sample *x*(*N*−*i*+1) and by the pattern (*x*(*N*−(*i*−1)+1),*x*(*N*−(*i*−2)+1), …, *x*(*N*−(*i*−*L*+1)+1)) containing *L*−1 future samples of *x*(*N*−*i*+1) and thus equivalent to $x_{L-1}^{\text{future}}(N-i+2)=(x(N-i+2),x(N-i+3),\ldots ,x(N-i+L))$. Therefore, the forward prediction of *x*^*r*^(*i*) based on *L*−1 past samples, $x_{L-1}^{r,\text{past}}(i-1)$, in *x*^*r*^ is equivalent to the backward prediction of *x*(*N*−*i*+1) based on *L*−1 future values, $x_{L-1}^{\text{future}}(N-i+2)$, in *x*.

Time irreversibility is captured by checking the changes in the statistical properties of the series occurring after time reversal.

### (b) Irreversibility index based on the sign of variations

Let us define Δ*x*^+^={Δ*x*(*i*)=*x*(*i*+1)−*x*(*i*)|Δ*x*(*i*)>0, *i*=1, …, *N*} and Δ*x*^−^={Δ*x*(*i*)=*x*(*i*+1)−*x*(*i*)|Δ*x*(*i*)<0, *i*=1, …, *N*} and let us indicate with *N*^+^ and *N*^−^ the number of Δ*x*(*i*) present in Δ*x*^+^ and Δ*x*^−^, respectively. Porta *et al*. (2008) suggested the assessment of irreversibility as *N*^−^ divided by *N*^+^+*N*^−^ (multiplied by 100). If Δ*x*(*i*)≠0 ∀*i*, *N*^+^+*N*^−^=*N*−1. This index will be termed as N% in the following. When the series is reversible, N% evaluated over *x* and *x*^*r*^ is approximately 50. A departure from 50 implies irreversibility since it indicates an imbalance between N% calculated over *x* and over *x*^*r*^ (i.e. 100−N% calculated over *x*). It is noteworthy that N%>50 implies that *N*^−^>*N*^+^, or, equivalently, that the averaged magnitude of |Δ*x*^+^| is larger than that of |Δ*x*^−^|, and, therefore, the distribution of Δ*x*=(Δ*x*(*i*)=*x*(*i*+1)−*x*(*i*), *i*=1, …, *N*−1) is skewed towards positive values. The reverse situation is observed with N%<50.

### (c) Irreversibility index based on local nonlinear prediction

Local nonlinear prediction (Farmer & Sidorowich 1987) assumes that, if the previous past values $\left(\text{e.g.}\;x_{L-1}^{\text{past}}(i-1)\;\text{and}\;x_{L-1}^{\text{past}}(j-1)\right)$ are similar, future values (i.e. *x*(*i*) and *x*(*j*)) will stay close. Here, the local nonlinear prediction approach is referred to as the local nonlinear forward prediction (LNLFP) approach to indicate that past values are used to predict ahead future values under the usual time evolution. The LNLFP technique will be differentiated from the local nonlinear backward prediction (LNLBP) approach that uses future values $\left(\text{i.e.}\;x_{L-1}^{\text{future}}(i+1)\right)$ to predict backward the current value *x*(*i*).

The LNLFP approach defines as the best prediction of *x*(*i*) based on *L*−1 previous samples, $x_{L-1}^{\text{past}}(i-1)$, a suitable statistics over all those *x*(*j*)'s such that their previous *L*−1 values, $x_{L-1}^{\text{past}}(j-1)$, are similar to $x_{L-1}^{\text{past}}(i-1)$. Here, the median is set as the best prediction of *x*(*i*) (i.e. zero-order predictor). In order to assess whether $x_{L-1}^{\text{past}}(j-1)$ is close to $x_{L-1}^{\text{past}}(i-1)$ or, in other words, if the pattern $x_{L-1}^{\text{past}}(j-1)$ is similar to $x_{L-1}^{\text{past}}(i-1)$, the signal *x* is uniformly quantized over *ξ*=6 bins (the resolution is (*x*_max_−*x*_min_)/*ξ*, where *x*_max_ and *x*_min_ are the maximum and the minimum of the series). Uniform quantization imposes a partition of the (*L*−1)-dimensional phase space into *ξ*^*L*−1^ disjoint hypercubes of the same size covering the entire phase space (Porta *et al*. 2007). The pattern $x_{L-1}^{\text{past}}(j-1)$ is similar (or close) to $x_{L-1}^{\text{past}}(i-1)$ if both stay inside the same hypercube. All the patterns inside a hypercube that contains $x_{L-1}^{\text{past}}(i-1)$ (including $x_{L-1}^{\text{past}}(i-1)$) contribute with their next values to the best prediction of *x*(*i*). The LNLBP approach can be derived simply by substituting $x_{L-1}^{\text{past}}(i-1)$ and $x_{L-1}^{\text{past}}(j-1)$ with $x_{L-1}^{\text{future}}(i+1)$ and $x_{L-1}^{\text{future}}(j+1)$. After defining the forward prediction error (FPE) as the difference between *x*(*i*) and its best prediction given past values $\hat{x}(i/x_{L-1}^{\text{past}}(i-1))$, the mean squared FPE (MSFPE) is used to evaluate the goodness of LNLFP at a given *L*. Analogously, the mean squared backward prediction error (MSBPE) is calculated by considering the difference between *x*(*i*) and its best prediction, given future values $\hat{x}(i/x_{L-1}^{\text{future}}(i+1))$. MSFPE and MSBPE at *L*=1 are set as the mean squared deviation from the median of *x* (MSD) representing the best prediction when no past (or future) samples are used. MSFPE=0 (or MSBPE=0) means perfect LNLFP (or LNLBP), while a large MSFPE (or MSBPE) means that prediction is poor.

The ‘in-sample’ procedure is used to evaluate the predictor (i.e. *x*(*i*) belongs to the same set of data used to calculate $\hat{x}(i/x_{L-1}^{\text{past}}(i-1))$ or $\hat{x}(i/x_{L-1}^{\text{future}}(i+1))$). We used the strategy proposed by Porta *et al*. (2000) to prevent overfitting, i.e. the overoptimistic progressive decrease in the prediction error, while increasing the number of past (or future) samples, *L*−1. Briefly, instead of using MSFPE to assess prediction we use the corrected MSFPE (CMSFPE) simply obtained by adding to MSFPE a corrective term defined as the product between MSD and the percentage of pattern $x_{L}^{\text{past}}(i)$ found alone in a hypercube (perc^past^(*L*)). Analogously, corrected MSBPE (CMSBPE) is calculated by considering perc^future^(*L*). Since the corrective terms, perc^past^(*L*) and perc^future^(*L*), grow monotonically with *L* towards MSD, CMSFPE and CMSBPE do not decrease towards 0 as MSFPE and MSBPE do, thus preventing overfitting. If the series is neither completely predictable nor fully unpredictable in forward and backward directions, these functions show a minimum over *L*. The minimum is taken as an unpredictability index and termed FUPI in the case of the CMSFPE minimum, and BUPI in the case of the CMSBPE minimum. Forward and backward predictions are not necessarily equal. If FUPI is smaller than BUPI, the series is more predictable in the forward direction than in the backward one. Even the position of the minimum can be different and indicates a difference between forward and backward prediction.

Simply by recalling the result given in appendix A*a*, LNLFP applied to *x*^*r*^ is equivalent to LNLBP applied to *x*. Therefore, the comparison of FUPI and BUPI derived from *x* is helpful to detect irreversibility based on prediction (i.e. to check the invariance of LNLFP after time reversal). For this purpose, we define FBUPI=(BUPI−FUPI)/(BUPI+FUPI). Positive values indicate that *x* is more predictable in the forward direction (i.e. using past values) than in the backward one (i.e. using future values). The index FBUPI is independent of the variance of the series.
